# Galectin-3 as a marker to characterize post-cardiac arrest syndrome in initially survived out-of-hospital cardiac arrest: a prospective two-center study

**DOI:** 10.1016/j.resplu.2025.101048

**Published:** 2025-07-30

**Authors:** Swantje Nickelsen, Eleonore Grosse Darrelmann, Lea Seidlmayer, Katrin Fink, Simone Britsch, Daniel Duerschmied, Ruediger E. Scharf, Albrecht Elsaesser, Thomas Helbing

**Affiliations:** a Department of General, Visceral and Transplant Surgery, Hannover Medical School, Carl-Neuberg-Straße-1, D-30625 Hannover, Germany; b Department of Cardiology, University Hospital Oldenburg, Carl von Ossietzky University, Rahel Straus Strasse 10, D-26133 Oldenburg, Germany; c University Emergency Center, Faculty of Medicine, University of Freiburg, Sir-Hans-A.-Krebs-Strasse, D-79106 Freiburg im Breisgau, Germany; d Center for Acute Cardiovascular Medicine Mannheim (ZKAM), Department of Cardiology, Angiology, Haemostaseology and Medical Intensive Care, University Medical Center Mannheim, Medical Faculty Mannheim, Heidelberg University, Theodor-Kutzner-Ufer 1-3, D-68167 Mannheim, Germany; e German Center for Cardiovascular Research (DZHK), Partner Site Heidelberg/Mannheim, Theodor-Kutzner-Ufer 1-3, D-68167 Mannheim, Germany; f European Center for Angioscience (ECAS), Medical Faculty Mannheim, Heidelberg University, Theodor-Kutzner-Ufer 1-3, D-68167 Mannheim, Germany; g Division of Experimental and Clinical Haemostasis, Haemotherapy, and Transfusion Medicine, and Haemophilia Comprehensive Care Center, Institute of Transplantation Diagnostics and Cell Therapy, Heinrich Heine University Medical Center, Moorenstraße 5, D-40225 Düsseldorf, Germany

**Keywords:** OHCA, Galectin-3, In-hospital mortality, PCAS, Cerebral edema, Shock, Interleukin-6

## Abstract

**Background:**

Survivors after out-of-hospital cardiac arrest (OHCA) experience post-cardiac arrest syndrome (PCAS), which encompasses cerebral edema, hemodynamic instability and systemic inflammation and causes high in-hospital mortality rates. Galectin (GAL) 3 is a predictor of mortality and unfavorable neurological outcome following OHCA. This study aims to investigate the relationship between GAL3 levels and key features of PCAS including in-hospital mortality, cerebral edema, post-cardiac arrest shock and systemic inflammation in OHCA patients.

**Methods:**

This prospective, two-center study included 71 adults after non-traumatic OHCA. Blood samples were taken on hospital admission (day 0) and day 2 after return of spontaneous circulation (ROSC). Serum GAL3 concentrations were quantified by enzyme-linked immunosorbent assay and compared with serum levels of 39 patients with coronary artery disease (CAD).

**Results:**

Serum GAL3 levels were highest on day 0 and declined on day 2 after ROSC to levels comparable to CAD controls. GAL3 levels were higher in non-survivors at both time- points. Admission GAL3 concentrations positively correlated with lactate on admission, a marker for no-flow/low-flow time and were elevated in patients with cerebral edema on cerebral computed tomography. Furthermore, admission GAL3 was higher in patients with inadequate lactate clearance and GAL3 levels on day 2 were significantly elevated in OHCA patients who required prolonged vasopressor/inotropic medication, both indicators of persistent hypoperfusion and shock. Moreover, a positive correlation was observed between GAL3 and interleukin-6 on admission.

**Conclusion:**

Serum GAL3 levels are associated with in-hospital mortality and distinct features of PCAS including cerebral edema, persistent shock and systemic inflammation following OHCA.

*German Clinical Trials Register No. DRKS00020250; DRKS00009684.*

## Highlights

• Following OHCA, GAL3 levels are elevated on day 0 and already normalized on day 2.
• Compared to survivors, GAL3 levels on admission are higher in OHCA non-survivors.
• Elevated GAL3 levels on day 0 are associated with brain edema in OHCA survivors.
• GAL3 levels on day 0 and 2 are associated with markers of shock in OHCA survivors.
• GAL3 levels are associated with IL-6 driven systemic inflammation after OHCA.

## Background

Out-of-hospital cardiac arrest (OHCA) is one of the leading causes of death in both Europe and the United States.^1^, ^2^, ^3^, ^4^, ^5^ Despite advances in management, the prognosis for patients who initially survive an OHCA remains poor.^1^, ^3^, ^4^, ^5^ Approximately 8 % of OHCA patients are discharged from hospital alive.^2^, ^3^, ^6^

The primary factor contributing to a poor outcome after return of spontaneous circulation (ROSC) is the development of post-cardiac arrest syndrome (PCAS). PCAS is characterized by hypoxic-ischemic encephalopathy (HIE), post-cardiac arrest myocardial dysfunction, hemodynamic instability and a systemic inflammatory response.^3^, ^7^ This syndrome arises as a consequence of whole-body ischemia/reperfusion (I/R) injury during prolonged resuscitation.^8^ The severity of PCAS is largely determined by the duration of global ischemia and the subsequent release of inflammatory mediators such as interleukins (IL) and reactive oxygen species during reperfusion.^1^

Galectin-3 (GAL3) is a 26 kDa β-galactosidase protein belonging to the family of β-galactoside binding lectins. It is expressed in inflammatory cells including neutrophils, monocytes and macrophages^9^, ^10^ and has been observed to play a role in the innate immune response.^9^, ^10^, ^11^, ^12^ Recently, two studies have demonstrated that admission serum GAL3 levels were higher in non-survivors and predicted short- and long-term mortality, as well as cerebral disability in post-cardiac arrest patients.^13^, ^14^ The role of GAL3 in the early phase after resuscitation remains unclear. This study explores the regulation of serum GAL3 at two time points in the early post-resuscitation phase and provides insight into the association between GAL3 levels and characteristics of post-cardiac arrest syndrome (PCAS).

## Methods

This is a prospective, two-center cohort study performed at the Department of Cardiology, University Hospital of Oldenburg, Germany and Department of Cardiology and Angiology at the University Hospital of Freiburg, Germany between May 2016 and December 2021. The protocol was approved by the ethics committee of the University of Oldenburg (No. 2020-021) and the University of Freiburg (No 239/16) and corresponds to the declaration of Helsinki. The study protocols for each center are available at the German Clinical Trials Register (DRKS00020250, DRKS00009684). Informed consent was obtained from resuscitated non-traumatic OHCA patients who survived to discharge with favorable neurological outcome or from their next of kin after personal contact. Upon admission to hospital, comatose OHCA patients who were older than 18 years and reached ROSC after ≥5 min of cardiopulmonary resuscitation (CPR) were eligible for inclusion. OHCA patients following trauma, pregnant patients and those with end-stage kidney disease (glomerular filtration rate <15 ml/min/1.73 m^2^) were excluded as impaired renal function increases GAL3 levels.^15^ One patient diagnosed with acquired immunodeficiency syndrome (AIDS) was excluded, as elevated GAL3 serum levels have been reported in human immunodeficiency virus (HIV) patients even when viral RNA is undetectable.^16^ In addition, this measure aimed to minimize any potential infection risk to blinded study personnel.

A control group of 39 patients with known coronary artery disease (CAD) was formed. As CAD patients are similar in age, sex and cardiovascular comorbidities to OHCA patients and CAD assessment requires coronary angiography, they form an adequate control group.^1^, ^17^, ^18^, ^19^ Information on patient management is provided within the supplement (Additional file 1).

### Outcome measures

The relationship between GAL3 levels and the following key features of PCAS was investigated: (1) in-hospital mortality, (2) signs of cerebral edema on cranial computed tomography (cCT), (3) lactate on admission, (4) duration of vasopressor and inotropic therapy (5) lactate clearance and (6) serum IL-6 levels.

Patients underwent cCT 48–72h after ROSC. cCT scans were evaluated for signs of cerebral edema by senior consultants of diagnostic radiology. The relationships between GAL3 and admission lactate levels and lactate clearance were also assessed. Inadequate lactate clearance was defined as a lactate concentration ≥2 mmol/L on day 2 after ROSC.^20^ To assess hemodynamic instability, the patients' need for different inotropic/vasopressor medications throughout the intensive care unit (ICU) stay was evaluated. To assess the relationship between GAL3 levels and the inflammatory response following OHCA, the extent of inflammation was measured by serum levels of IL-6.^21^, ^22^, ^23^, ^24^, ^25^, ^26^, ^27^

### Blood sampling and biomarker measurements

For patients with OHCA, the first venous blood sample was collected from a venous catheter within 0–6h after ROSC in the intensive care unit (day 0). To assess GAL3 regulation during the intermediate phase of PCAS – beyond the immediate effects of ischemia/reperfusion injury (day 0)^1^ – follow-up blood sampling was routinely performed on day 2 as part of the early morning laboratory work-up in the ICU, typically between 5:00 and 6:00 a.m. NSE was measured at individual timepoints for each patient at 48h after ROSC to ensure optimal timing for neuroprognostication.^3^ A cut-off of >60 ng/ml is considered indicative of poor neurological outcome.^3^ In CAD controls, blood samples were collected by research staff immediately following coronary angiography. After collection and centrifugation, the isolated serum was immediately stored at −21 °C until it was shipped to the central laboratory for ELISA measurements. Serum GAL3 was measured using the Quantikine ELISA Human Galectin-3 Immunoassay (DGAL30, USA R&D Systems Inc., Minneapolis, MN, USA). IL-6 and NSE were measured by the respective hospitals’ Department of Clinical Chemistry and Laboratory Medicine. Further information is provided in Additional file 2.

### Statistical analyses

Continuous variables were tested for normal distribution with Shapiro-Wilk’s test and are presented as median and interquartile range (IQR). Continuous variables were compared using Mann-Whitney-*U* test or Wilcoxon signed-rank test. Categorical variables are presented as count and percentages and differences were tested with the chi-square (Chi^2^) test or Fisher’s exact test. Correlations between metric variables were analyzed by Spearman’s rank correlation and the coefficient rho (*r*) is reported.

The serial determination of GAL3 within each patient allowed for analyzing associations with dichotomous outcome variables by repeated measures linear mixed models. GAL 3 was the dependent variable. Fixed effects included time, the respective outcome measure and their interaction term while patients were included as a random effect. The model was estimated using REML. Model results were reported after checking for underlying assumptions (normal distribution of residuals, homoscedasticity). For each model, post-hoc pairwise comparisons were conducted using Šidák’s test to differentiate if differences in GAL3 with regard to the respective outcome variable could be attributed to a specific time point of GAL3 measurement. To assess the value of GAL3 for outcome prediction, receiver operating characteristic (ROC) analyses were performed. Results are reported as area under curve (AUC). The optimal cut-off was determined with the maximized Youden index. The optimal cut-off value was then included in a univariate logistic regression model as an independent categorical predictor. Results are reported as Odds Ratio (OR) with 95 % CI. All tests were two-sided and statistical significance was defined as *p* < 0.05 unless otherwise specified. Statistical analyses were performed using IBM SPSS Statistics 29 (IBM, Armonk, NY, USA). Plotting and mixed models were done with GraphPad Prism 10 (GraphPad Software, San Diego, CA, USA).

## Results

Seventy-one patients after non-traumatic OHCA were included for analyses. Details on patient in- and exclusion are provided in the supplement (Additional file 3). Patient characteristics are shown in Table 1. The average age was 64 (IQR 55.8–74.5) years and 71.8 % (*n* = 51) of patients were male. Median estimated no-flow time was 2.5 (IQR 0.0–10.0) min and median time to ROSC was 20.0 (IQR 15.0–30.5) min. Shockable rhythms were observed in 52.1 % (*n* = 37) of OHCA patients. In-hospital mortality was 60.6 % (*n* = 43). The median time to death was 3 (IQR 1–7) days.

**Table 1:** Baseline characteristics and survival status after OHCA.

	Survivors	Non-survivors	*p*
	*n* = 28	*n* = 43	
Age [years], median (IQR)	63.0 (48.5–75.5)	66.0 (60.0–81.0)	0.063
Male sex, *n* (%)	22 (75.6)	29 (67.4)	0.420
Comorbidities, *n* (%)			
Coronary artery disease	19 (67.9)	18 (41.8)	0.052
Arterial hypertension	16 (57.1)	25 (58.1)	1.000
Chronic heart failure	5 (17.9)	4 (9.3)	0.469
Diabetes mellitus	3 (10.7)	8 (18.6)	0.506
COPD	1 (3.6)	5 (11.6)	0.389
Cardiac cause of OHCA, *n* (%)	23 (82.1)	23 (53.5)	**0.039**
First documented rhythm, *n* (%)			**<0.001**
Shockable rhythm	22 (75.6)	15 (34.9)	
Asystole	1 (3.6)	18 (41.8)	
Pulseless electrical activity	5 (17.9)	10 (23.3)	
Time to CPR (no-flow time) [min], median (IQR)	1.0 (0.0–2.0)	6 (1.0–10.0)	**0.002**
Time to ROSC [min], median (IQR)	15.0 (12.0–25.0)	20 (10.0–40.0)	0.267
Left ventricular dysfunction on admission, *n* (%)			1.000
LVEF ≤30 %	9 (32.1)	13 (30.2)	
LVEF >30 %	14 (50.0)	22 (51.2)	
No echocardiography available	5 (17.9)	8 (18.6)	
ECLS, *n* (%)	3 (10.7)	6 (14.0)	1.000
Application of vasopressors, *n* (%)	27 (96.4)	38 (88.4)	0.641
Application of inotropics, *n* (%)	9 (32.1)	14 (32.6)	1.000
Vasopressors/inotropics >3 days, *n* (%)	15 (53.6)	16 (37.2)	0.325
Lactate on admission [mmol/L], median (IQR)	4.3 (2.0–6.3)	7.4 (4.0–12.0)	**0.001**
Adequate lactate clearance, *n* (%)	19 (67.9)	11 (25.6)	**0.009**
Cause of death	/		
Hypoxic-ischemic encephalopathy, *n* (%)		26	
Cardiovascular, *n* (%)		11	
Other (e.g. sepsis, respiratory), *n* (%)		6	
CPC			**<0.001**
CPC 1–2, *n* (%)	20 (71.4)	0 (0)	
CPC 3–5, *n* (%)	8 (28.6)	43 (100.0)	
NSE 48h [ng/ml], median (IQR)*	47.4 (29.0–90.8)	67.4 (27.0–180.0)	0.361
cCT obtained, *n* (%)	23 (79.3)	38 (88.4)	0.323
TTM to 33 °C for 24 h, *n* (%)	23 (82.1)	31 (72.1)	0.765

Data are presented as median and IQR. Survival status is based on survival to hospital discharge. The *p*-value represents comparison between groups. *p* < 0.05 was considered significant.

*CAD* coronary artery disease, *cCT* cranial computed tomography, *COPD* chronic obstructive pulmonary disease, *CPC* cerebral performance category, *CPR* cardiopulmonary resuscitation, *ECLS* extracorporeal life support*, IQR* interquartile range*, LVEF* left ventricular ejection fraction*,* min minutes, *NSE* neuron-specific enolase, *OHCA* out-of-hospital cardiac arrest, *ROSC* return of spontaneous circulation*, TTM* targeted temperature management.

* ECLS patients were excluded from this analysis.

**Fig. 1:**
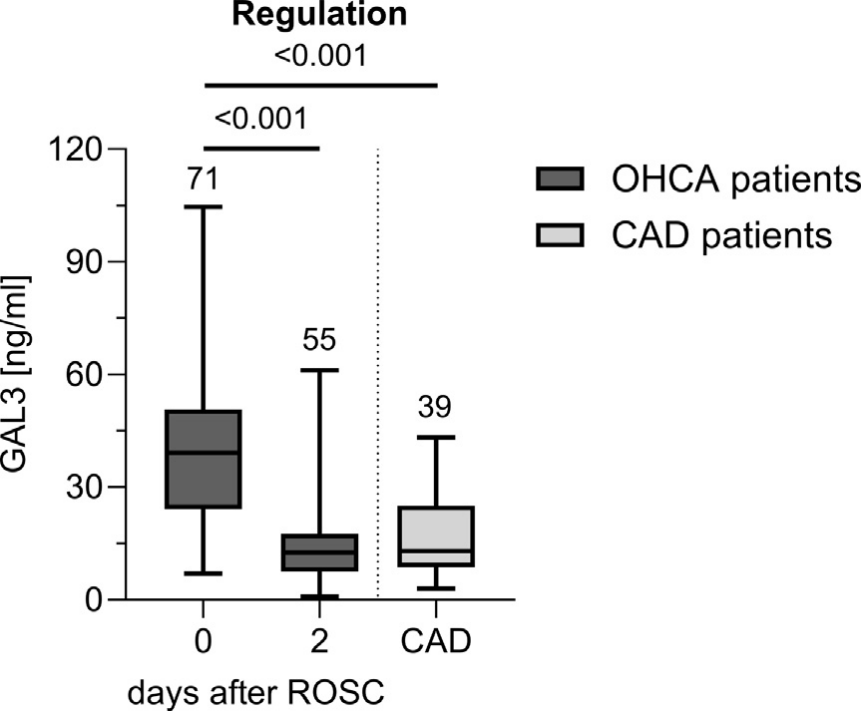
*Regulation of serum GAL3 levels in patients after OHCA.* Concentrations of GAL3 were highest on admission and showed a significant decrease within 48h after ROSC. Boxplots show median with IQR, whiskers denote range. Digits above whiskers show number of patients in each group. Between-day differences tested with Wilcoxon signed-rank test. Mann-Whitney-*U* test performed for between-group comparison. *p* < 0.05 was considered significant. *CAD* coronary artery disease, *GAL3* Galectin-3, *IQR* interquartile range, *OHCA* out-of-hospital cardiac arrest, *ROSC* return of spontaneous circulation.

**Fig. 2:**
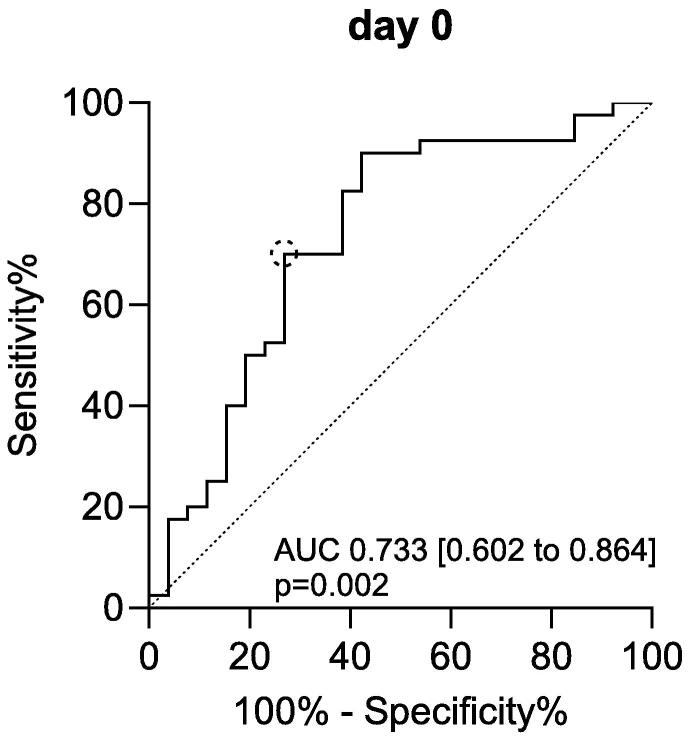
*GAL3 levels on admission are higher in non-survivors after OHCA.* ROC-analysis shows good classification for GAL3 levels on admission. Statistics of analysis given within the figure. To determine the optimal cut-off which maximizes sensitivity and specificity, Youden’s Index was calculated (YI = 0.43, shown as dotted circle). *p* < 0.05 was considered significant. *AUC* area under curve, *GAL3* Galectin-3, *OHCA* out-of-hospital cardiac arrest, *ROC* receiver-operating characteristic.

Serum GAL3 levels on admission (day 0) were significantly higher than on day 2 after ROSC (*p* < 0.001; Fig. 1, Table 2). GAL3 levels at the time of hospital admission were significantly higher than in CAD controls, while GAL3 levels on day 2 were comparable to those measured in the CAD group (Fig. 1, Table 2). GAL3 levels showed significant differences between survivors and non-survivors on admission (*p* = 0.015; Table 2). A cut-off of GAL3 on day 0 > 37.48 ng/ml was identified to predict death with a sensitivity of 70.0 % and a specificity of 73.1 % (Univariate logistic regression analysis: OR 9.45 [2.60–34.34], *p* < 0.001; Fig. 2).

**Table 2:** Serum concentrations of GAL3 for OHCA patients and CAD controls.

	GAL3 [ng/ml], median (IQR)		
	OHCA (*n* = 71)		CAD (*n* = 39)
	day 0	day 2	
Total	39.19 (24.10–50.78)	12.63 (7.53–17.54)	13.0 (8.72–25.01)
Survival status			
Survivors (*n* = 28)	24.13 (15.89–42.47)	10.02 (5.71–15.54)	13.0 (8.72–25.01)
Non-survivors (*n* = 43*)	43.88 (31.31–57.77)	16.13 (9.98–21.35)	/
p**	**0.015**	0.079	

Šidák’s test was used to control for multiple testing; *p* < 0.05 was considered significant. *CAD* coronary artery disease, *GAL3* Galectin-3, *IQR* interquartile range, *OHCA* out-of-hospital cardiac arrest.

* Number of patients on day 0 (day of admission); non-survivors day 2 *n* = 27.

** The presented *p*-values are results of post-hoc multiple comparison tests on linear mixed models.

GAL3 levels on admission were significantly higher in OHCA patients with cerebral edema on cCT (*p* = 0.019; Table 3), which represents the morphological correlate of HIE and reflects the extent of cerebral injury following I/R. Median blood lactate level on admission was 5.6 (IQR 2.2–7.7) mmol/L and decreased on day 2 to 1.7 (IQR 1.1–3.1) mmol/L. Serum GAL3 levels at admission showed a positive correlation to lactate on admission (*r* = 0.276, *p* = 0.036), a surrogate marker of no-flow and low-flow time during CPR.

**Table 3:** Serum levels of GAL3 and key features of PCAS.

	GAL3 [ng/ml], median (IQR)	
	day 0	day 2
Brain edema on cCT		
No brain edema (*n* = 44)	39.18 (24.24–48.79)	14.03 (7.07–21.52)
Brain edema (*n* = 16)	50.21 (27.72–101.93)	12.32 (6.89–17.66)
p*	**0.019**	0.678
Duration of vasopressor/inotropic support		
≤3 days (*n* = 22)	40.98 (25.06–50.23)	10.60 (4.43–15.95)
>3 days (*n* = 31)	31.23 (23.84–59.79)	13.98 (9.03–21.95)
p*	0.996	**0.030**
Lactate clearance		
Adequate (*n* = 30)	31.23 (23.84–21.49)	10.09 (7.14–16.49)
Inadequate (*n* = 25)	41.36 (21.10–73.02)	14.07 (9.03–21.38)
p*	**0.014**	0.419

Šidák’s test was used to control for multiple testing; *p* < 0.05 was considered significant. *cCT* cerebral computed tomography, *GAL3* Galectin-3, *IQR* interquartile range, *OHCA* out-of-hospital cardiac arrest, *PCAS* post-cardiac arrest syndrome.

* The presented *p*-values are results of post-hoc multiple comparison tests on linear mixed models.

31.0 % (*n* = 22) of OHCA patients required vasopressor/inotropic medication for a maximum duration of three days (≤3 days), whereas 43.7 % (*n* = 31) of patients required such therapy for at least four days (>3 days) throughout the post-resuscitation period (Table 3). GAL3 levels on day 2 were significantly elevated in patients who required prolonged vasopressor/inotropic support in comparison to those who needed shorter vasopressor/inotropic support for a period of ≤3 days (*p* = 0.030; Table 3).

Adequate lactate clearance was observed in 42.3 % (*n* = 30) patients after OHCA, while 35.2 % (*n* = 25) of patients showed lactate ≥2 mmol/L on day 2 after ROSC (inadequate lactate clearance). As an indicator of hemodynamic stabilization, adequate lactate clearance was associated with survival to hospital discharge in logistic regression analyses (OR 4.44 [1.41–13.97], *p* = 0.011). Serum GAL3 levels at admission were significantly higher in patients with inadequate lactate clearance, indicating that GAL3 levels are higher in OHCA patients with hemodynamic instability (*p* = 0.014; Table 3).

In this OHCA cohort, IL-6 was already elevated on admission and increased further on day 2 after OHCA (day 0: 120.8 [IQR 34.6–321.1] ng/L; day 2: 200.1 [95.7–592.9] ng/L). A positive correlation was observed between GAL3 on day 0 and IL-6 on admission but not on day 2 (day 0: *r* = 0.393, *p* = 0.001; day 2: *r* = 0.133, *p* = 0.378)*.*

## Discussion

This study presents novel findings on the regulation of serum GAL3 levels in the early phase after ROSC and its diagnostic and prognostic value after initial OHCA survival. Key observations include: First, serum GAL3 levels on hospital admission after OHCA are significantly higher than both levels measured on day 2 and those observed in patients with CAD (Fig. 1). Second, elevated serum GAL3 levels on hospital admission are a predictor of in-hospital mortality after OHCA (Fig. 2). Third, GAL3 levels on admission are elevated in OHCA patients with cerebral edema on cCT, the morphological correlate of hypoxic-ischemic encephalopathy and showed a positive correlation with lactate on admission (Table 3). Fourth, higher GAL3 levels are associated with hypoperfusion and post-cardiac arrest shock reflected by prolonged need of vasopressor/inotropic support and inadequate lactate clearance (Table 3). Fifth, GAL3 on admission correlates with IL-6 on admission, an independent predictor of mortality and PCAS severity.^22^, ^23^, ^27^, ^28^

Upregulation of GAL3 has been observed in a variety of critically-ill patients on ICU presenting with sepsis, trauma or COVID-19.^29^, ^30^, ^31^ In addition, elevation of GAL3 was reported in patients with severe heart failure with need for mechanical circulatory support and acute myocardial infarction (AMI) with hemodynamic instability.^32^, ^33^ Two studies have previously revealed GAL3 elevation on hospital admission in serum of OHCA patients.^13^, ^14^ The present study is the first to analyze the temporal profile of GAL3 beyond admission during the early post-resuscitation period (Fig. 1). GAL3 levels peaked on admission after OHCA and declined on day 2 to levels comparable to those observed in CAD controls suggesting that GAL3 down-regulation is linked to whole-body reperfusion. This pattern aligns with findings in stroke and ST-elevation myocardial infarction patients, where GAL3 levels peaked during ischemia and declined after reperfusion.^34^, ^35^ Similarly, in our OHCA cohort, the initial GAL3 peak likely reflects no-flow/low-flow ischemia during resuscitation, while the subsequent decrease indicates reperfusion. Supporting this, patients with persistent ischemia from post-cardiac arrest shock showed higher GAL3 levels on day 2, along with ongoing vasopressor use and impaired lactate clearance (Table 3).^36^, ^37^, ^38^

Overall outcome prediction remains a central challenge during the early post-resuscitation phase.^3^ Two previous studies have suggested a possible prognostic role of admission GAL3 levels in OHCA.^13^, ^14^ In one prospective multicenter study, serum GAL3 levels at hospital admission following OHCA were higher in non-survivors and concentrations >26.6 ng/ml were identified as an independent risk factor for all-cause mortality after 4 weeks as well as 5 months of follow-up.^13^ A recent study has indicated that elevated GAL3 levels (>26.3 ng/ml) at the time of admission can serve as a predictor of mortality after 5.7 years in OHCA survivors.^14^ The present study identified an optimal cut-off value to predict in-hospital mortality of ≥37.48 ng/ml (Fig. 2). Therefore, our results are in line with the available literature suggesting that GAL3 levels on admission may serve as a potential biomarker that might contribute to prognostication after OHCA.

The majority of fatalities after OHCA are attributed to HIE.^1^, ^39^, ^40^ After initial stabilization of an OHCA patient, a multimodal algorithm to evaluate the extent of HIE is recommended by the European Resuscitation Council (ERC) guidelines.^3^ In the present study, GAL3 on admission demonstrated neither an association with NSE after 48h, nor was there a correlation with neurological outcomes as determined by Cerebral Performance Categories (CPC) at the time of hospital discharge (Additional files 4 and 5). However, serum GAL3 levels were found to correlate with the presence of cerebral edema as depicted on cCT and with lactate on admission, which has been previously identified as a predictor of poor neurological outcome.^20^, ^36^, ^41^, ^42^ A recent analysis has identified GAL3 on admission as a predictor of long-term cerebral disability assessed by CPC at 5.7 years after OHCA.^14^

Post-resuscitation shock occurs in up to 60 % of OHCA patients, contributes to multi-organ failure and poor prognosis and requires early and aggressive management.^3^, ^39^, ^40^, ^43^ The complex pathophysiology of post-resuscitation shock includes vasoplegia and myocardial dysfunction due to systemic I/R injury and sepsis-like syndrome, both features of post-cardiac arrest syndrome (PCAS).^1^, ^3^ In this cohort, GAL3 levels remained higher in patients with prolonged vasopressor requirements and inadequate lactate clearance on day 2 after ROSC (Table 3). This identifies GAL3 as a novel potential marker of prolonged hypoperfusion and shock after cardiac arrest. Supporting this findings, GAL3 concentrations were observed to correlate with need for inotropic medication in acute heart failure, AMI and a cohort of patients after left-ventricular assist device implantation.^33^, ^44^, ^45^

In recent years, evidence on lactate clearance as a surrogate parameter for resolution of hypoperfusion and shock has emerged.^36^ Interpretation of a singular lactate value is difficult as lactate levels are representative of both lactate build-up due to e.g. tissue hypoxia during resuscitation but are also influenced by post-cardiac arrest aspects that hinder lactate elimination processes.^36^, ^37^, ^38^ Lactate clearance within 3–48h after admission predicts short-term and 30-day survival following ROSC.^20^, ^37^, ^46^, ^47^, ^48^, ^49^ In line with these reports, this study shows that adequate lactate clearance is associated with survival to hospital discharge and GAL3 levels are higher in patients with inadequate lactate clearance (Table 3).

The early post-resuscitation phase involves a systemic inflammatory response with elevated cytokines such as IL-6 and TNF-α.^21^, ^50^, ^51^ In this cohort, IL-6 increased on day 2. Elevated IL-6 within 72h post-ROSC has been linked to organ dysfunction, PCAS severity and higher mortality,^22^, ^23^, ^27^, ^28^ as well as endothelial activation and vasopressor need.^24^, ^25^ IL-6 is induced via toll-like receptor (TLR) signaling^52^ and GAL3 may contribute by activating TLR4, thereby promoting IL-6 production.^53^ In this study, admission GAL3 correlated with IL-6 on day 0, supporting its role in triggering inflammation via innate immune activation.^12^, ^54^ GAL3 enhances neutrophil function and macrophage recruitment and polarization after ischemic injury.^12^ These observations, in line with previous studies, suggest a possible involvement of GAL3 in the inflammatory response following cardiac arrest.

### Limitations

Despite the prospective, two-center study design, the present study has several limitations and the results should be applied carefully to other situations and populations. Due to early mortality, not all outcome measures could be assessed in the entire cohort (*n* = 71). Specifically, brain edema could be evaluated in 60 patients, lactate clearance in 55 patients, and the need for prolonged vasopressor or inotropic support in 53 patients (Additional file 6). These missing data may introduce selection bias and should be considered when interpreting the subgroup results. The proportion of survivors (39 %) in this study is considerably higher than reported in international studies and the German resuscitation register with survival rates below 20 %.^1^, ^2^, ^39^, ^55^ The rather low mortality can be attributed, at least in part, to the high percentage of patients with shockable rhythms (52.1 %) and cardiac cause (64.8 %) as a trigger for cardiac arrest which is known to be associated with better outcome.^56^ A number of patients died after life-sustaining therapy was withdrawn for poor neurological prognosis after neuroprognostication in line with ERC guidelines. These patients cannot be differentiated from cases that fulfilled brain death criteria.

As is inherit to any observational study, we cannot prove a causal relationship but only describe the association between GAL3 and outcome parameters. It cannot be excluded that, at least in some of the patients, GAL3 levels were already elevated before OHCA due to pre-existing comorbidities. In this cohort, subgroup analyses show that GAL3 levels are higher in non-survivors with prior ischemic heart disease, arterial hypertension and diabetes mellitus (data not shown). However, the respective number of patients analyzed in each subgroup was small, hence, these results should be re-evaluated in larger patient settings. Most of the resuscitated patients were undergoing targeted temperature management (TTM). We cannot exclude possible effects of TTM on GAL3 levels.

## Conclusions

The present study demonstrates upregulation of GAL3 in the post-resuscitation period. GAL3 levels on admission are a possible predictor of in-hospital mortality after OHCA and are associated with cerebral edema and IL-6 driven inflammation. Moreover, elevated GAL3 levels were associated with ongoing post-cardiac arrest shock.

## CRediT authorship contribution statement

**Swantje Nickelsen:** Writing – review & editing, Writing – original draft, Visualization, Investigation, Formal analysis, Data curation. **Eleonore Grosse Darrelmann:** Writing – review & editing, Resources, Investigation. **Lea Seidlmayer:** Writing – review & editing, Resources, Investigation. **Katrin Fink:** Writing – review & editing, Conceptualization. **Simone Britsch:** Writing – review & editing, Supervision. **Daniel Duerschmied:** Writing – review & editing, Supervision. **Ruediger E. Scharf:** Writing – review & editing, Supervision. **Albrecht Elsaesser:** Visualization, Conceptualization. **Thomas Helbing:** Writing – review & editing, Writing – original draft, Visualization, Project administration, Funding acquisition, Conceptualization.

## Funding

This work was supported by intramural funds to S.N. and T.H. through the University Oldenburg (FP 2020-052_Helbing) and by the DFG (HE 7432/1-1). The funding source was not involved in study design; the collection, analyses and interpretation of data; in the writing of the report and in the decision to submit the article for publication.

## Declaration of competing interest

The authors declare the following financial interests/personal relationships which may be considered as potential competing interests: “D. Duerschmied is supported by the DFG (CRC1366 B08, project number 394046768, and CRC1425 P07, project number 422681845) and the DZHK (MaBo-05).”.
