# Supplementary material for: Galectin-3 as a marker to characterize post-cardiac arrest syndrome in initially survived out-of-hospital cardiac arrest: a prospective two-center study
Source: Resusc Plus. 2025 Jul 30;25:101048. doi: 10.1016/j.resplu.2025.101048 (PMC12357278; doi:10.1016/j.resplu.2025.101048)
Supplement: Supplementary Data 1 [file mmc1.pdf]

Additional file 1 Patient management

The centers at Oldenburg and Freiburg recruited 25 and 46 patients, respectively. All patients were admitted to the medical ICU. Invasive hemodynamic monitoring of patients was performed with a pressure catheter in the radial artery. Crystalloid fluids were administered in all patients to achieve general treatment goals for central venous pressure (CVP) of 10-15 mmHg to optimize right heart filling pressure and urine output >1.5 ml/kg/h. The hemodynamic target was mean arterial pressure >65 mmHg to ensure sufficient organ perfusion. Vasopressor/inotropic agents were used if volume substitution alone did not result in adequate hemodynamics or was not feasible. Percutaneous coronary intervention (PCI) was performed if a patient presented with ST-segment elevation on electrocardiogram (ECG) or at the treating physician's discretion. Targeted temperature management (TTM) after ROSC was applied according to ERC guidelines at the time of patient inclusion [1]. Withdrawal of life-sustaining treatment decisions for poor neurological outcome were made by senior consultants of neurology and intensive care medicine in accordance with the ERC guideline recommendations [1].

Additional file 2 Blood sampling and biomarker measurements

After sample collection and centrifugation, the isolated serum was stored at -21°C until it was shipped to the central laboratory, where it was further stored at -80°C. At both centres, samples were thawed immediately before enzyme-linked immunosorbent assay (ELISA) measurements and were not subjected to repeated freeze-thaw cycles. Serum GAL3 was measured using the Quantikine ELISA Human Galectin-3 Immunoassay (DGAL30, USA R&D Systems Inc., Minneapolis, MN, USA). Intra-assay and inter-assay precision were 3.8% and 6.3%, respectively. Analyses were performed according to the manufacturer's protocol and personnel were blinded to all clinical information. All samples were analyzed in duplicate.

Additional file 3 Study flow diagram

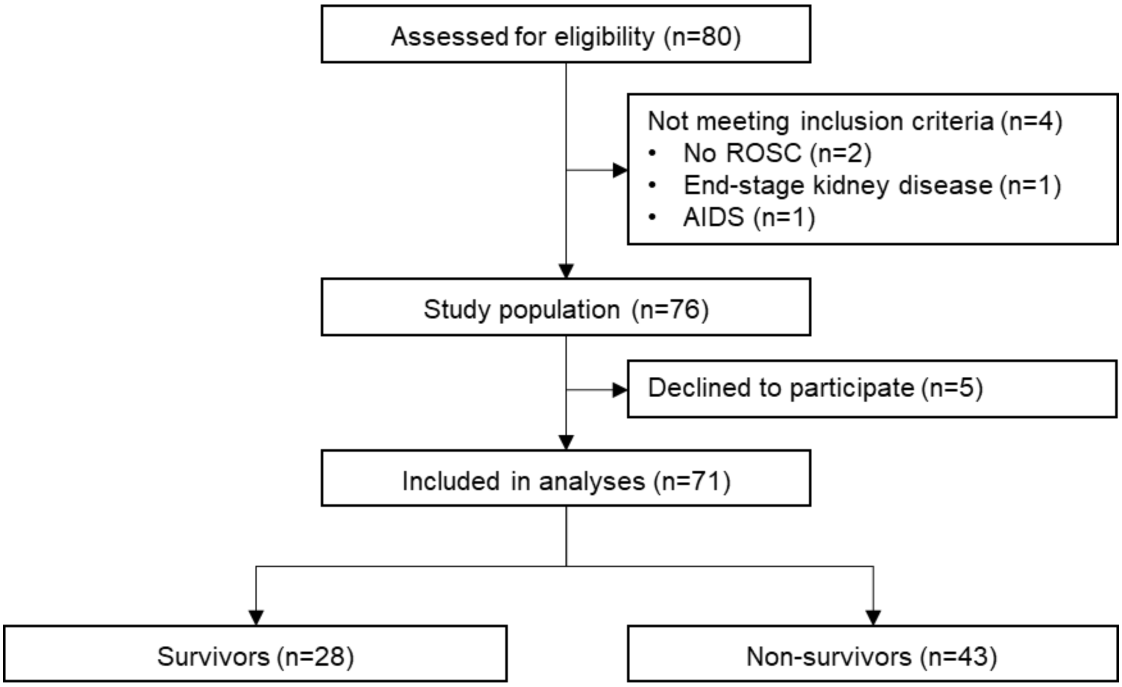

Legend: Flowchart of the study population available for analyses. The AIDS patient was excluded to prevent confounding due to elevated GAL3 levels in HIV, even in the absence of detectable viral RNA. AIDS acquired immune deficiency syndrome, *GAL3* galectin-3, *HIV* human immunodeficiency virus, *ROSC* return of spontaneous circulation

Additional file 4 Correlation of GAL3 on admission and NSE at 48h in patients after OHCA

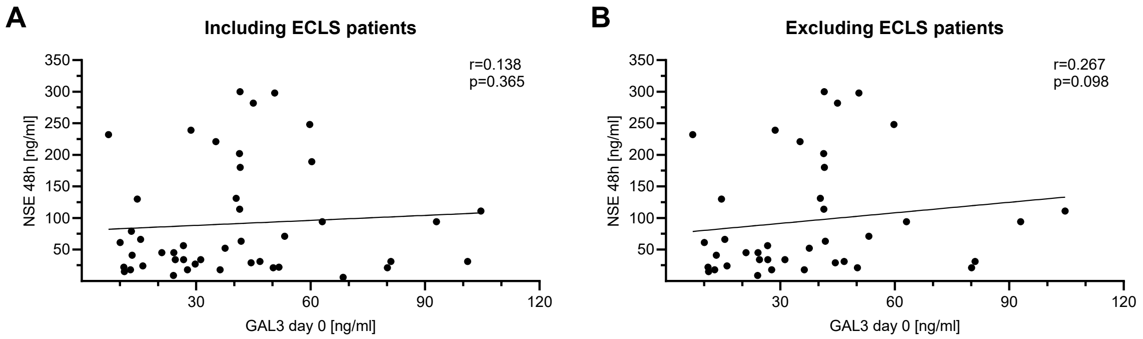

Legend: GAL3 on admission did not correlate with NSE at 48h after ROSC (A). When ECLS patients were excluded to account for potential confounding by hemolysis, a trend towards a significant correlation between admission GAL3 and NSE after 48h was observed (B). Results of Spearman correlation coefficient r and respective p-value are presented within the figure.

p<0.05 was considered significant. *ECLS* extra-corporal life support, *GAL3* galectin-3, *IQR* interquartile range, *NSE* neuron-specific enolase, *OHCA* out-of-hospital cardiac arrest,

Additional file 5 GAL3 and neurological outcome in patients after OHCA

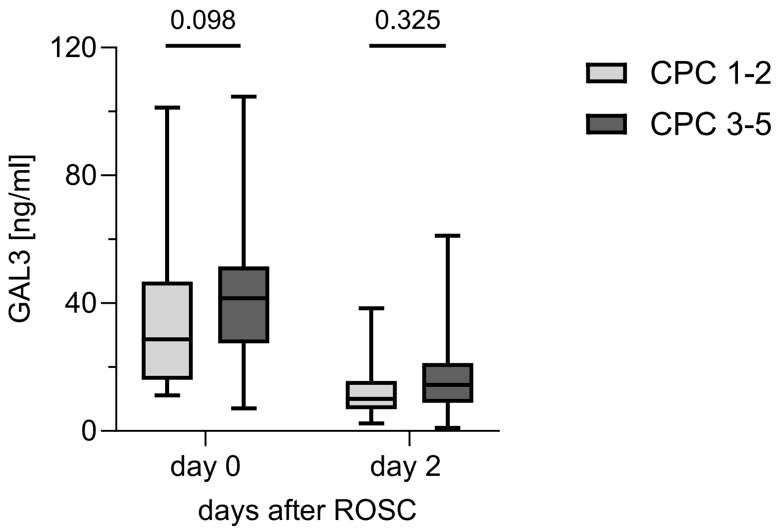

Legend: GAL3 was not significantly higher in patients with poor neurological outcome. Boxplots show median with IQR, whiskers denote range. The presented p-values are results of post-hoc multiple comparison tests on linear mixed models and Šidák's test was used to control for multiple testing; p<0.05 was considered significant. CPC cerebral performance category, GAL3 galectin-3, IQR interquartile range, OHCA out-of-hospital cardiac arrest

Additional file 6 Missing data

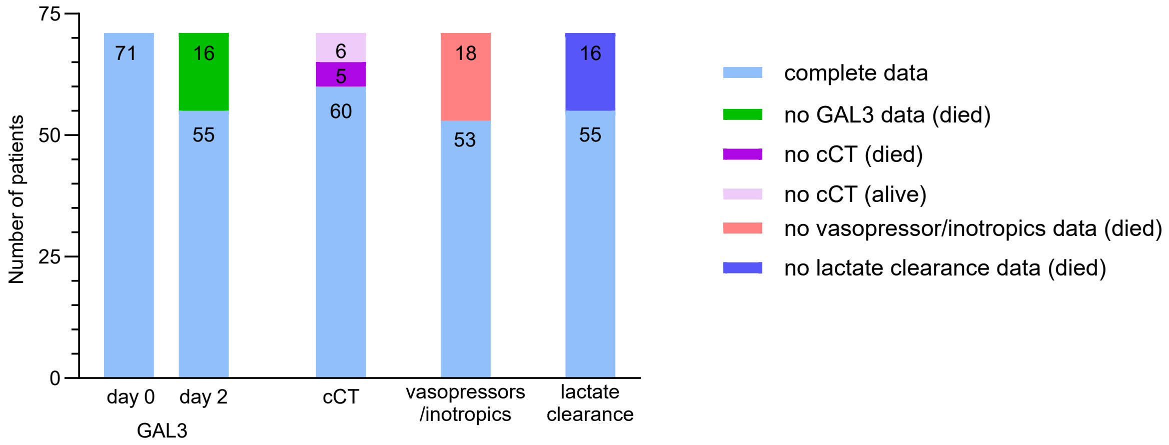

Legend: Bar chart illustrating the number of patients available for analyses for each outcome measure. The exact number of patients is given within the bar. *cCT* cranial computed tomography, *GAL3* galectin-3

References

1. Nolan JP, Sandroni C, Böttiger BW, Cariou A, Cronberg T, Friberg H, et al. European Resuscitation Council and European Society of Intensive Care Medicine Guidelines 2021: Post-resuscitation care. Resuscitation. 2021;161:220-69.
